# PDC: a highly compact file format to store protein 3D coordinates

**DOI:** 10.1093/database/baad018

**Published:** 2023-04-03

**Authors:** Chengxin Zhang, Anna Marie Pyle

**Affiliations:** Department of Computational Medicine and Bioinformatics, University of Michigan, 100 Washtenaw Av, Ann Arbor, MI 48109, USA; Howard Hughes Medical Institute, 4000 Jones Bridge Rd, Chevy Chase, MD 20815, USA; Department of Molecular, Cellular, and Developmental Biology, Yale University, 266 Whitney Av, New Haven, CT 06511, USA; Howard Hughes Medical Institute, 4000 Jones Bridge Rd, Chevy Chase, MD 20815, USA; Department of Molecular, Cellular, and Developmental Biology, Yale University, 266 Whitney Av, New Haven, CT 06511, USA; Department of Chemistry, Yale University, 225 Prospect St, New Haven, CT 06511, USA

## Abstract

Recent improvements in computational and experimental techniques for obtaining protein structures have resulted in an explosion of 3D coordinate data. To cope with the ever-increasing sizes of structure databases, this work proposes the Protein Data Compression (PDC) format, which compresses coordinates and temperature factors of full-atomic and Cα-only protein structures. Without loss of precision, PDC results in 69% to 78% smaller file sizes than Protein Data Bank (PDB) and macromolecular Crystallographic Information File (mmCIF) files with standard GZIP compression. It uses ∼60% less space than existing compression algorithms specific to macromolecular structures. PDC optionally performs lossy compression with minimal sacrifice of precision, which allows reduction of file sizes by another 79%. Conversion between PDC, mmCIF and PDB formats is typically achieved within 0.02 s. The compactness and fast reading/writing speed of PDC make it valuable for storage and analysis of large quantity of tertiary structural data.

**Database URL** https://github.com/kad-ecoli/pdc

## Introduction

Due to powerful new experimental structure determination methods and the maturation of highly accurate protein structure prediction pipelines, such as AlphaFold (1), RoseTTaFold (2) and Distance-based Iterative Threading ASSEmbly Refinement (D-I-TASSER) (3), many protein structures that were previously unattainable at high accuracy are now available as models on public databases such as Protein Data Bank (PDB) and the AlphaFold Protein Structure Database (AlphaFold DB) (4). For example, while the AlphaFold DB only had 50 gigabytes of protein structure models in macromolecular Crystallographic Information File (mmCIF) and PDB formats in 2021, it hosts 470 times more predicted protein structures (23 terabytes) in 2022. In the future, the size of this database is expected to keep increasing. The rapid accumulation of structural data will make it increasingly difficult for research laboratories to store and analyze these data. Therefore, a file format that can more efficiently store the same structural information within limited disk space is needed.

As the main file formats for storing macromolecular structure information, the mmCIF (also known as PDBx) and PDB formats were designed with legibility rather than file size in mind. There are two main reasons for their large file sizes. First, mmCIF and PDB files have many information redundancies. For example, the type and index of a residue are repeated several times in a file, once for each atom in the residue. Second, both mmCIF and PDB formats are text files, which are not efficient in storing floating-point numbers. For example, the 3D coordinates of an atom are stored as three eight-character strings of text rather than three floating-point numbers in binary, although an eight-character string takes up 8 bytes while a floating-point number takes up only 4 bytes. Moreover, there are many white spaces used to designate different fields in the file, further increasing the file size. The standard procedure to reduce file size of mmCIF and PDB files by the PDB and AlphaFold database is to apply the general-purpose GZIP compression. While GZIP is efficient in eliminating redundancies in a text file, it is not specifically developed for coordinate data and therefore only offers a limited degree of compression.

To more effectively compress and store macromolecular structure information, several new file formats have been previously proposed. For example, the BinaryCIF (5) format aims to store all information of an mmCIF file in a binary format, which enables more efficient storage and parsing. The Macromolecular Transmission Format (MMTF) (6) format is another binary format, which was specifically developed by the Research Collaboratory for Structural Bioinformatics Protein Data Bank database to reduce the size of coordinate files. Both BinaryCIF and MMTF mainly perform lossless compression, where the precision of coordinates and temperature factors (down to 0.001 Å and 0.01 Å^2^, respectively) are not compromised after compression. In addition to lossless compression, some implementations of MMTF also enable lossy compression by retaining only one digit after decimal. On the other hand, the PIC (7) format only performs lossy compression with a slight loss of precision (usually ∼0.1 Å) by applying the Portable Network Graphics (PNG) compression algorithm for atom positions in the spherical coordinate space. Although PNG is a lossless compression algorithm, the coordinate conversion from Cartesian to spherical space introduces rounding error effects, which makes PIC compression lossy.

This work proposes the Protein Data Compression (PDC) format, an even more space-efficient file format to compress protein structures from the AlphaFold DB. Compared to general structure files such as those from PDB, protein structure models from the AlphaFold DB have several unique characteristics that warrant a more specific data compression format. First, the AlphaFold-predicted models do not have missing atoms, alternatively located atoms or heteroatoms, allowing the omission of certain data fields such as atomic types, alternative location indicators and occupancies. Second, since the bond lengths and bond angles in the AlphaFold models are all near ideal, the structural information can be stored in torsion space rather than Cartesian space for lossy compression without visually perceptible differences. Third, predicted structure models lack several data fields not directly related to coordinate deposition, such as secondary structures and disulfide bonds. This allows further shrinking of file sizes. While the highly specific file format allows efficient storage by PDC, it also means that PDC is not meant to replace more general formats such as MMTF and BinaryCIF for experimental structures, which can have heteroatoms and missing/duplicated atoms.

## Methods

### Lossless compression by PDC

**Figure 1.**
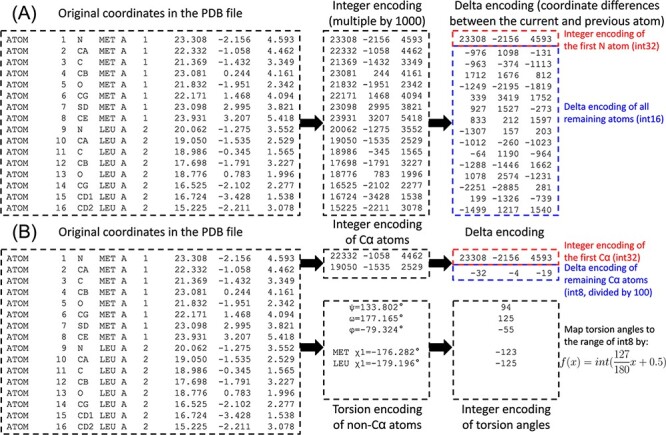
Illustration of PDC compression of the coordinates for the first two residues for the AlphaFold structure of methylated-DNA—protein-cysteine methyltransferase (UniProt ID P0AFH0). (A) Lossless compression. (B) Lossy compression.

Compression of mmCIF or PDB format protein structure files into PDC format is performed in three stages: integer encoding, delta encoding and data packing (Figure 1A). In the first stage, since mmCIF and PDB files store 3D coordinates and temperature factors with only 3 and 2 digits after decimal, respectively, they can be perfectly encoded by integers. In AlphaFold models, the temperature factors are in the range of 0.00 to 100.00. After multiplying by 100, they are within the range of 2-byte (i.e. 16 bit) integers, which have a range of −32 768 to 32 767. On the other hand, 3D coordinates in the PDB files are in the range of −999.000 to 9999.000. After multiplying by 1000, they are within the range of 4-byte (i.e. 32 bit) integers, which range from −2 147 483 648 to 2 147 483 647.

Although the coordinates of a given atom can span a large range, the difference in coordinates between two sequentially adjacent atoms is much smaller. For example, the carboxyl C atom of a residue and the amino N atom of the next residue are ∼1.3 Å apart. Therefore, in the second stage, ‘delta encoding’, which was originally proposed for the MMTF format (8), is performed by converting coordinates to differences in coordinates. To this end, atoms within each residue are reordered by proximity to the backbone (Table 1). The coordinate differences between an atom and the previous atoms can then be consistently accurate within each residue. For the first atom (N) in each residue, the C atom of the previous residue is considered the ‘previous’ atom to calculate the coordinate difference. In this way, the original coordinates represented by 4-byte integers can be compressed into 2-byte integers for coordinate differences.

**Table 1.** Atomic order and number of side chain torsions for different amino acid types

Residue type	Atomic order ^a^	Side chain torsion angles
GLY	N CA C O	None
ALA	N CA C CB O	None
CYS	N CA C CB O SG	χ1
ASP	N CA C CB O CG OD1 OD2	χ1 χ2
GLU	N CA C CB O CG CD OE1 OE2	χ1 χ2 χ3
PHE	N CA C CB O CG CD1 CD2 CE1 CE2 CZ	χ1 χ2
HIS	N CA C CB O CG CD2 ND1 CE1 NE2	χ1 χ2
ILE	N CA C CB O CG1 CG2 CD1	χ1 χ2
LYS	N CA C CB O CG CD CE NZ	χ1 χ2 χ3 χ4
LEU	N CA C CB O CG CD1 CD2	χ1 χ2
MET	N CA C CB O CG SD CE	χ1 χ2 χ3
ASN	N CA C CB O CG ND2 OD1	χ1 χ2
PRO	N CA C CB O CG CD	χ1 χ2
GLN	N CA C CB O CG CD NE2 OE1	χ1 χ2 χ3
ARG	N CA C CB O CG CD NE NH1 NH2 CZ	χ1 χ2 χ3 χ4 χ5
SER	N CA C CB O OG	χ1
THR	N CA C CB O CG2 OG1	χ1
VAL	N CA C CB O CG1 CG2	χ1
TRP	N CA C CB O CG CD1 CD2 CE2 CE3 NE1 CH2 CZ2 CZ3	χ1 χ2
TYR	N CA C CB O CG CD1 CD2 CE1 CE2 OH CZ	χ1 χ2

a For the last amino acid in a chain, the OXT atom is added as the last atom.

After integer encoding and delta encoding, difference data fields of the structure are packed into a single binary file. Different from mmCIF and PDB files where each line is all the information designating each atom, the PDC file consolidates the same types of data together to minimize text redundancies. The data types packed into a PDC file include:

- Title
- Compound: molecule name and chain ID
- Source: scientific name and National Center for Biotechnology Information taxonomy ID of the organism
- Database reference: UniProt accession code, UniProt entry ID and the range of sequence index that the protein structure model maps to the UniProt sequence
- One line for chain ID, the ‘B-factor mode’ and the sequence length
- Protein sequence (one-letter code)
- Residue indices. Continuous residue indices are recoded as ranges rather than a list of individual residues, i.e. as ‘1∼10’ rather than 1, 2, 3, 4, 5, 6, 7, 8, 9, 10 for ten residues with consecutive residue indices.
- Coordinates of the first N atom (4-byte integers)
- Coordinate differences of all remaining atoms (2-byte integers)
- Temperature factors (2-byte integers).

Here, ‘B-factor mode’ refers to repetitions of temperature factors. B-factor mode = 0 means that all atoms in the structure have the same value, and therefore, only value is recorded in the temperature factor field. B-factor mode = 1 means that all atoms in the same residue have the same temperature factor, while different residues have different temperature factors. The temperature factor field of the PDC file will include the same number of values as the number of residues. This is the most common case of AlphaFold structure models. B-factor mode = 2 means that each atom has its own temperature, all of which needs to be stored in the PDC file. During PDC file generation, the B-factor mode is automatically inferred from the input mmCIF or PDB file.

### Lossy compression by PDC

While PDC by default performs lossless compression, it can optionally perform lossy compression by small sacrifice of precision (Figure 1B). Lossy compression mode of PDC starts with calculating the coordinate differences between sequentially adjacent Cα atoms. Since adjacent atoms are separated by ∼3.8 Å, or 3800 in integer encoding, the differences in integer coordinates after division by 100 fall within the range of 1-byte integers (−128 to 127). Meanwhile, the φ, ψ and ω backbone torsion angles and χ1 to χ5 side chain torsion angles of each residue are calculated. The exact number of side chain torsion angles to calculate is detailed in Table 1 and follows the definition by the Dunbrack Rotamer Library (9). Each torsion angle $x$ is then mapped to the range of 1-byte integers by $f\left( x \right) = int\left( {\frac{{127}}{{180}}x + 0.5} \right)$, where $int$ means downward rounding. Differences in temperature factors are also calculated between adjacent Cα atoms, as well as (in the case of B-factor mode 2) between other atoms in the same residue and the Cα atom. These differences are multiplied by 10 and converted to 1-byte integers. When packing data for a lossy PDC file, the first seven data types are identical, while the remaining data types are modified as follows:

- Coordinates of the first Cα atom (4-byte integers)
- Coordinate differences of all remaining Cα atoms (1-byte integers)
- ϕ, ψ and ω torsion angles of backbone (1-byte integers)
- χ angles of side chains (1-byte integers)
- Temperature factor of the first Cα atom (2-byte integers)
- Temperature factor differences for remaining Cα atoms (1-byte integers)
- (B-factor mode 2 only) Temperature factor differences for non-Cα atom (1-byte integers).

To decode a lossy PDC back into an mmCIF or a PDB format file, the Cartesian coordinates of Cα atoms are first recovered from Data Fields 8 and 9. Meanwhile, the backbone and side chain torsion angles are read from Fields 12 and 13 and used by a C++ reimplementation of the PeptideBuilder (10) algorithm to reconstruct the full-atomic structure of the protein in torsion angle space. Full-atomic torsion space protein is fragmented into three-residue fragments and superimposed onto the Cα-only Cartesian space structure by least square fit (11). The combination of torsion and Cartesian space structures takes full advantage of the small size of torsion space representation while avoiding small inaccuracy in torsion angles or small deviations from ideal bond lengths or ideal bond angles, resulting in large impact on the global structure. While there are previous studies (12, 13) for reduced representation of protein and nucleic acid structures in the torsion space, PDC is the first algorithm to combine Cartesian and torsion space representations to achieve high fidelity structure compression.

## Results

### Datasets

The lossless and lossy modes of PDC compression are compared to the original mmCIF and PDB format files as well as three existing macromolecular structure compression schemes (BinaryCIF, MMTF and PIC) on the *Escherichia coli* subset of the AlphaFold DB from 2022. For MMTF, both the default lossless mode and the lossy mode are tested. The dataset consists of 4363 protein structure models in mmCIF and PDB formats ranging from 16 to 2358 residues. The performance of different compression algorithms is measured by file size in kilobytes (kb) and the time to compress and decompress a structure (in seconds). Since both the original mmCIF and PDB files from the AlphaFold DB and the PDC files generated by the PDC compressor apply GZIP compression, GZIP compression is applied to BinaryCIF, MMTF and PIC files before file size measurements for the sake of fairness.

In addition to this *E. coli* dataset, another dataset for human proteins is also prepared to investigate the impact of protein structure modeling methods on lossy compression accuracy. This dataset is generated by the common set of 19 205 proteins available in both the AlphaFold DB and the HPmod database (https://zhanggroup.org/HPmod/) of D-I-TASSER (3)-predicted structures. While AlphaFold-predicted structures only contain heavy atoms, D-I-TASSER-predicted structures also contain hydrogen. To make the benchmark result on AlphaFold and D-I-TASSER comparable to each other, only hydrogens are excluded. The datasets are available at https://doi.org/10.5281/zenodo.7554830.

### Overall performance of compression algorithms

**Figure 2.**
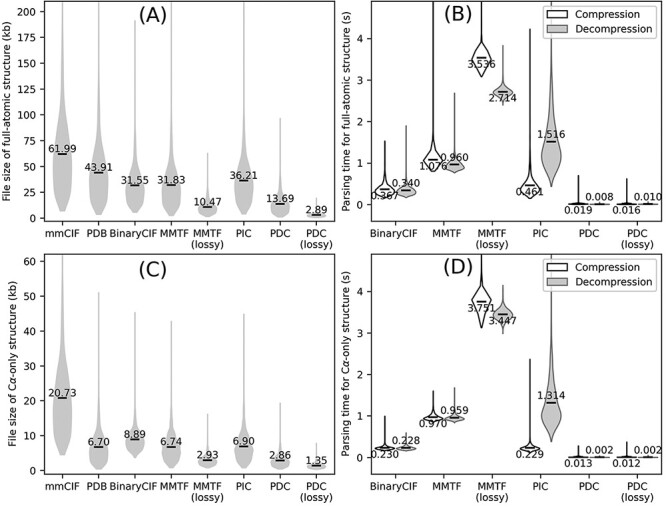
Overall performance of different structure compression schemes on the AlphaFold DB *E. coli* dataset. (A–B) File sizes (A) and compression/decompression time (B) for full-atomic structures. (C–D) File sizes (C) and compression/decompression time for Cα-only structures. Conversion to/from BinaryCIF, MMTF and PIC was performed by python-modelcif, Atomium and PIC, respectively. Conversion to/from lossy MMTF was performed by BioJava. Conversion of mmCIF versus PDB files to PIC files leads to different PIC metadata files; this figure uses the PIC files converted from PDB files because the resulting PIC files are smaller in size. The markers and values the violin plots indicate the average values for each method.

We first tested the PDC and three existing compression algorithms on full-atomic protein *E. coli* structures (Figure 2A and B). Even in lossless mode, PDC results in 56.6%, 57.0% and 62.2% smaller file size compared to all three existing methods (BinaryCIF, MMTF and PIC, respectively) with one-tailed paired *t*-test *P*-values <1E-303 for all three comparisons. Its average file size is also significantly smaller than the original mmCIF and PDB files by 77.9% and 68.8%, respectively, with *t*-test *P*-values <1E-303. Among the three existing compression algorithms, PIC has the largest file size. This is because PIC only compresses the coordinates in the structure files, while leaving other information such as temperature factors, residue names and atom names as unprocessed metadata. This leads to worse overall compression rate by PIC compared to BinaryCIF and MMTF despite more efficient (albeit lossy) compression of coordinates. Indeed, without considering metadata, the average file sizes of PDC-compressed coordinates are 14.85 kb, which is comparable to the size of PDC files (13.69 kb). Among the tested compression algorithms, the only existing algorithm producing smaller file size to PDC lossless compression is the MMTF lossy compression, whose average file size is 23.5% smaller than PDC lossless compression but 3.6 times larger than PDC lossy compression.

A practically useful file format should be fast to read and write. This study measures the parsing speed of a compressed file format by the time to convert an mmCIF file to the compressed file format (compression) and to convert the compressed file back to mmCIF file (decompression). Both compression and decompression are performed on 64 bit Red Hat Enterprise Linux 7.9 with a single CPU core (Intel Xeon Gold 6226 CPU, 2.70 GHz). Compression and decompression for PDC take on average 0.019 and 0.016 s per file, which are 19.3 and 42.5 times faster than the next fastest compression format (BinaryCIF) (Figure 2B). The speed of parsing a specific file format depends on the implementation of the file parsing program and not necessarily represents the superiority or inferiority of a file format itself. In any case, this benchmark shows that PDC parsing can be completed with negligible time.

In addition to compressing full-atomic structures, a PDC file can also store Cα-only structures (Figure 2C and D). In these cases, the coordinate of the first Cα atoms and the coordinate differences of all remaining Cα atoms are kept, while coordinates and torsion angles for other atoms are discarded. Cα-only structures are particularly useful for protein structure alignments, where many alignment programs only need Cα information (14, 15). Structure compression algorithms developed previously were not specifically optimized for Cα-only structures, where BinaryCIF, MMTF and PIC all have slightly larger file sizes than PDB files (Figure 2C). On the other hand, PDC can effectively compress Cα-only structures, resulting in reductions of file sizes by 86.2% and 57.3% compared to mmCIF and PDB formats, respectively, with significant *P*-values <1E-303 (Figure 2C).

### Lossy versus lossless compression

**Figure 3.**
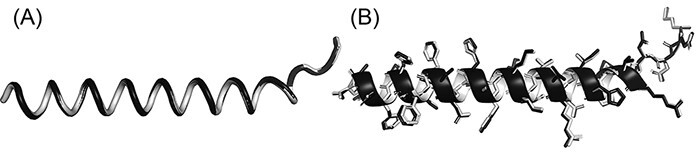
Superimposition between the original structure (black) and after PDC lossy compression (white). (A) Cα structure. (B) Full-atomic structure of Leader peptide SpeFL from *E. coli* (UniProt ID: P0DTV7), which is the protein with the worst Cα MAE (Cα MAE = 0.104 Å; non-Cα MAE = 0.193 Å) among all proteins in the *E. coli* benchmark dataset (average MAE = 0.094 and 0.167 Å for Cα and non-Cα atoms, respectively).

Some biological applications tolerate slight inaccuracies in the structures, such as in visualization of global topology and in detection of templates for structure-based function annotation (16). This is why PDC includes the lossy compression mode to achieve even smaller compressed file size with small sacrifice in coordinate precision. On average, the file sizes of lossy compression are only 78.9% and 47.2% of lossless PDC files for full-atomic and Cα-only structures, respectively (Figure 2A and C), with similar file reading/writing speeds (Figure 2B and D). On average, the mean absolute error (MAE) of coordinates resulting from lossy compression is 0.094 and 0.167 Å for Cα and non-Cα atoms, respectively. Although these MAE values are larger than those of PIC and MMTF lossy compression (Table 2), they are still small enough to be visually imperceptible (Figure 3).

The MAE of PDC lossy compression of predicted structures is affected by the structure prediction pipelines. For example, for the same set of 19 261 human proteins, lossy PDC compression results in MAE values of 0.095 and 0.250 for Cα and non-Cα atoms, respectively, for AlphaFold-predicted structures but 0.167 and 0.318 for D-I-TASSER-predicted structures (Table 2). This is partly because PDC lossy compression assumes near ideal bond lengths and bond angles, which can be assumed for the AlphaFold pipeline, where the molecular dynamics step at the end of the pipeline performs a more thorough refinement of local geometry of the structure models than the D-I-TASSER pipeline.

**Table 2.** Average performance of different compression methods on full-atomic structures of *E. coli* and human proteins

Dataset	Metric	BinaryCIF	MMTF	MMTF (lossy)	PIC	PDC	PDC (lossy)
*E. coli* (AlphaFold)	File size (kb)	31.55	31.83	10.47	36.21	13.69	2.89
	Compression time (s)	0.367	1.076	3.536	0.461	0.019	0.016
	Decompression time (s)	0.340	0.960	2.714	1.516	0.008	0.010
	Cα MAE (Å)	0	0	0.048	0.030	0	0.094
	Non-Cα MAE (Å)	0	0	0.048	0.030	0	0.167
Human (AlphaFold)	File size (kb)	44.91	47.57	13.63	52.24	20.27	4.18
	Compression time (s)	0.404	0.779	3.567	0.731	0.037	0.037
	Decompression time (s)	0.463	0.943	2.575	2.503	0.014	0.020
	Cα MAE (Å)	0	0	0.048	0.034	0	0.095
	Non-Cα MAE (Å)	0	0	0.048	0.034	0	0.250
Human (D-I-TASSER)	File size (kb)	38.78	45.42	12.84	48.76	19.42	3.66
	Compression time (s)	0.382	0.967	3.663	0.666	0.027	0.026
	Decompression time (s)	0.184	1.003	2.437	2.012	0.017	0.018
	Cα MAE (Å)	0	0	0.048	0.033	0	0.167
	Non-Cα MAE (Å)	0	0	0.048	0.033	0	0.318

## Conclusion

This work presents the highly compact PDC format to store the coordinate and temperature factor information of protein structures in binary format. A large-scale benchmark shows that delta encoding at lossless mode and combination of Cartesian and torsion space representations at lossy mode enables more effective compression.

The PDC format was originally designed to parse AlphaFold-predicted protein structure models. To generalize PDC on other macromolecular structures in the future, several modifications will be needed, including the marking of missing atoms and addition of dedicated fields for heteroatoms.

## Data Availability

The C++ source code of the compression and decompression programs to convert mmCIF and PDB files to and from PDC files is available at https://github.com/kad-ecoli/pdc under the BSD license. All structural files needed to reproduce this work are available at https://doi.org/10.5281/zenodo.7554830.
